# Classification of Cognitive Level of Patients with Leukoaraiosis on the Basis of Linear and Non-Linear Functional Connectivity

**DOI:** 10.3389/fneur.2017.00002

**Published:** 2017-01-19

**Authors:** Ranran Li, Youzhi Lai, Yumei Zhang, Li Yao, Xia Wu

**Affiliations:** ^1^College of Information Science and Technology, Beijing Normal University, Beijing, China; ^2^Neurology Department, Beijing Tiantan Hospital Affiliated with Capital Medical University, Beijing, China; ^3^State Key Laboratory of Cognitive Neuroscience and Learning, Beijing Normal University, Beijing, China

**Keywords:** leukoaraiosis, functional connectivity, eMIC, fMRI, cognitive level classification

## Abstract

Leukoaraiosis (LA) describes diffuse white matter abnormalities apparent in computed tomography (CT) or magnetic resonance (MR) brain scans. Patients with LA generally show varying degrees of cognitive impairment, which can be classified as cognitively normal (CN), mild cognitive impairment (MCI), and dementia. However, a consistent relationship between the degree of LA and the level of cognitive impairment has not yet been established. We used functional magnetic resonance imaging (fMRI) to explore possible neuroimaging biomarkers for classification of cognitive level in LA. Functional connectivity (FC) between brain regions was calculated using Pearson’s correlation coefficient (PCC), maximal information coefficient (MIC), and extended maximal information coefficient (eMIC). Next, FCs with high discriminative power for different cognitive levels in LA were used as features for classification based on support vector machine. CN and MCI were classified with accuracies of 75.0, 61.9, and 91.1% based on features from PCC, MIC, and eMIC, respectively. MCI and dementia were classified with accuracies of 80.1, 86.2, and 87.4% based on features from PCC, MIC, and eMIC, respectively. CN and dementia were classified with accuracies of 80.1, 89.9, and 94.4% based on features from PCC, MIC, and eMIC, respectively. Our results suggest that features extracted from fMRI were efficient for classification of cognitive impairment level in LA, especially, when features were based on a non-linear method (eMIC).

## Introduction

Leukoaraiosis (LA), also called cerebral white matter hyperintensities, is evidenced by decreased density on CT and increased signal intensity on T2/FLAIR sequences performed as part of MRI brain scans. LA mainly manifests in non-specific changes in the ventricle or subcortical white matter. These changes have been regarded as an important symptom of cerebral ischemia (1–3). Previous studies have suggested that LA leads to cognitive impairment mainly manifested in thinking ability, memory and executive function, attention ability, and cortical cognitive dysfunction (4, 5). However, the term LA is derived from neuroimaging studies and describes structural changes in the brain. Several studies have shown that the degree of LA does not consistently predict cognitive impairment (6, 7). For example, many patients have been diagnosed with LA but did not show any associated clinical abnormality (8, 9). Analyzing cognitive impairment levels of patients with LA and comparing cognitive impairment levels with functional imaging data could be helpful in identifying neuroimaging biomarkers for the different cognitive impairment levels associated with LA.

Blood oxygen level dependent (BOLD) based functional magnetic resonance imaging (fMRI) is a non-invasive technique measuring hemodynamic changes caused by neural activity. Neural activity levels in specific brain regions are detected indirectly by monitoring changes of deoxygenated hemoglobin concentration in the local blood stream (10, 11). fMRI has been widely used to study the operational organization of the human brain and is useful to study LA (12–15). A recent study has shown that the amplitude of low frequency fluctuations of signals measured in resting-state fMRI in brain regions of the right inferior occipital gyrus, left middle temporal gyrus, left precuneus, right superior frontal gyrus, and right superior occipital gyrus significantly differed among patients showing LA with cognitive impairment, patients showing LA without cognitive impairment, and normal individuals (12). Functional connectivity (FC) has been defined as the temporal correlation of one neurophysiological index measured in multiple brain regions and has been employed to characterize interactions between brain regions (16–19). FC has been critical for the study of functional interaction between different brain regions. Linear FC has been used to study the working mechanism of brain, aiding us to further our understanding of how different brain regions coordinate (20–22). Previous studies have shown that the FC between the posterior cingulate cortex and medial prefrontal frontal cortex of the default mode network is altered in LA [e.g., Ref. (23)]. Moreover, FC among the right cingulate motor area, left posterior insula, and left ventral premotor area has been shown to be significantly decreased in LA (13, 24). Abnormal FC owing to LA has been shown to be associated with several forms of cognitive impairment (25, 26), Therefore, FC estimated based on resting-state fMRI can be used to study the potential of FC to serve as a predictor for cognitive impairment level in patients with LA, including cognitively normal (CN), mild cognitive impairment (MCI), and dementia.

As a linear measure of the association between a pair of random variables, Pearson’s correlation coefficient (PCC) has been widely used to study FC between brain regions (27). However, linear correlation measures of the connectivity between brain regions may not be suited to capture the complex interaction between brain regions (28–30). Along these lines, BOLD signal has been shown to have non-linear properties (31, 32). Therefore, non-linear FC might be better suited to capture complex interactions between brain regions in patients with LA using fMRI. Maximal information coefficient (MIC) has been proposed as a new tool to measure the association of two time variables (33) and has been suggested to be an appropriate method to reconstruct the brain functional network (34). The extended maximal information coefficient (eMIC; a combination of MIC and PCC) measures non-linear associations between two variables (33, 35). eMIC has been employed to estimate non-linear FC in a non-linear connectivity analysis of schizophrenia. This previous study has shown that non-linear FC had discriminative power in the diagnosis of schizophrenia (36). The authors suggested that non-linear FC might provide crucial information for disease identification.

In this study, we aimed to investigate the changes in FC among three groups of patients with LA showing different levels of cognitive impairment (CN, MCI, and dementia). We aimed to study the discriminative power of linear and non-linear FC for different cognitive impairment levels based on the features of linear and non-linear FC. Discriminative power was measured using Kendall tau coefficient. FCs with high discriminative power were used as features for support vector machine (SVM) to comprehensively classify cognitive levels associated with LA. SVM is based on statistical learning theory and is widely used for classification, prediction, and pattern recognition tasks (37–40).

## Materials and Methods

### Subjects

Fifty patients with LA were involved in this study: 21 patients with CN (average age, 58.8 years; 10 female and 11 male), 16 with MCI (average age, 64.2 years; 10 female and 6 male), and 13 with dementia (average age, 65.0 years; 5 female and 8 male). Subjects were grouped based on the scores of the mini-mental state examination (MMSE). Patients with MMSE scores from 28 to 30 were classified as CN, scores from 23 to 27 were classified as MCI, and scores smaller than 23 were classified as dementia (41). There was no significant difference in age among the three groups (Table 1). The entire study including fMRI data acquisition, clinical imaging diagnosis of LA, and cognitive performance assessment was performed at the Tiantan Hospital, Beijing, China. All subjects were right-handed, without mental or brain disease other than vascular dementia, and without a history of cognitive or antipsychotic medication. All experimental protocols were approved by the institutional review board of Tiantan Hospital, and all subjects signed informed consent forms.

**Table 1:** **Demographic and clinical characteristics of subjects**.

	CN	MCI	Dementia	pValue	pValue	pValue

				CN-MCI	CN-dementia	MCI-dementia
Number of subjects (female/male)	10/11	10/6	5/8	0.368^a^	0.601^a^	0.198^a^
Age (mean ± SD)	58.8 ± 8.80	64.2 ± 10.97	65.0 ± 13.33	0.127^b^	0.166^b^	0.867^b^
MMSE (mean ± SD)	28.9 ± 0.72	26.3 ± 0.98	20.5 ± 2.34	0.00^b^	0.00^b^	0.00^b^
MoCA (mean ± SD)	24.6 ± 2.90	20.6 ± 2.80	15.1 ± 3.63	0.00^b^	0.00^b^	0.00^b^

*CN, cognitively normal; MCI, mild cognitive impairment; MMSE, mini-mental state examination; MoCA, Montreal Cognitive Assessment*.

*^a^The *p-*value was obtained by two-sample chi-square (χ^2^) test*.

*^b^The p-value was obtained by two-sample independent samples t-test*.

### Data Acquisition

Functional Magnetic Resonance Imaging data were collected using a 3-T Siemens whole-body MRI system at the Tiantan Hospital. Subjects were asked to keep heads still, eyes closed, and stay awake during scans. Resting-state functional scans were acquired with a T2*-weighted echo-planar imaging sequence, with specific scanning parameters: echo time, 30 ms; repetition time, 2,480 ms; flip angle, 90°, 36 axial slices in each volume; field of view, 256 mm × 256 mm; matrix size, 64 × 64; slice thickness, 3 mm, and voxel size = 3 mm × 3 mm × 4 mm. A total of 240 volumes were collected (9 min and 55 s in total) for each subject.

### Data Preprocessing

Data were preprocessed using SPM8 software.^1^ First, for each subject, the first 10 volumes were discarded to allow participants to adapt to the circumstances. Second, 230 rest volumes were preprocessed using slice-timing to adjust for differences in image acquisition time between slices, all other slices were corrected to the middle slice. Third, motion correction was performed. Head motion of none of the subjects exceeded the 2 mm or 2° range. Therefore, all subjects were included. Fourth, images were normalized to the Montreal Neurological Institute template (42) and resampled to a 3 mm × 3 mm × 3 mm per voxel resolution. Finally, normalized data were smoothed using a Gaussian kernel with a full width at half maximum of 6 mm.

### FC Matrix Establishment

Preprocessed data from fMRI scans were divided into 116 brain regions according to the automatic anatomical labeling (AAL) template, which was generated by WFU_PickAtlas toolkit.^2^ Each brain region was represented by the average of all voxels in the brain region area, transforming the fMRI data for each subject into a 116 × 230 matrix, where 116 corresponds to the number of brain regions and 230 corresponds to the length of the time series. The value of each point was the average of all the voxel values in the brain region at the corresponding moment. The brain FC of each participant was calculated separately using PCC, MIC, and eMIC, giving three FC matrixes for each participant. FC values between every pair of brain regions were defined as features.

Pearson’s correlation coefficient between two variables was calculated as the ratio between the covariance of the two variables and the product of their standard deviations, which is described as:

(1) $$\rho_{XY}=\frac{cov\left(X,Y\right)}{\sigma_{X}\sigma_{Y}}=\frac{E\left[\left(X-\mu_{X}\right)\left(Y-\mu_{Y}\right)\right]}{\sigma_{X}\sigma_{Y}}$$

To calculate the MIC (33) between two variables, a grid *G* (with *x* rows and *y* columns) was drawn on the scatterplot of the two-variable data set. It partitioned the data to capture the relationship between the two subsets. Mutual information was maximized over all *x*-by-*y G*s applied to the data set (up to a maximal grid resolution depending on sample size). The mutual information of two random variables was defined as follows:

(2) $$\begin{array}{l} \begin{array}{ll} I\left(X,Y\right) & =\;H\left(X\right)+H\left(Y\right)-H\left(X,Y\right)\\ & =\;H\left(X\right)-H\left(X\text{|}Y\right)\\ & =\;H\left(Y\right)-H(Y|X) \end{array} \end{array}$$

where *H*(*X*) and *H*(*Y*) are the entropies of *X* and *Y*, respectively, *H*(*X*|*Y*) and *H*(*Y*|*X*) are the conditional entropies, and *H*(*X*,*Y*) is the joint entropy of *X* and *Y*. *I_G_* was defined as the mutual information of the probability distribution partitioned by *G*. To allow comparison of grids of different resolutions, I*_G_* was normalized to [0,1]. Thus, the *m_x_*_×_*_ y_* of the characteristic matrix was defined as:

(3) $$m_{x\times y}=\frac{{\text{max}}_{g\in G_{x\times \text{y}}}\left\{I_{G}\right\}}{\text{log min}\{(x,y)\}}$$

MIC is the maximum *m_x_*_×_*_y_* over all ordered pairs (*x*,*y*) and *xy* < *B*, with *B* = *n*^0.6^, with *n* corresponding to the length of the vectors. Thus, MIC was defined as:

(4) $$MIC{\text{ = max}}_{xy\text{<}B}\{m_{x\times y}\}$$

eMIC (33) is a measure of the non-linear correlation between two variables *X* and *Y*, which can be simply described as:

(5) $$\text{eMIC}=\text{MIC}-\rho^{2}$$

where ρ corresponds to the PCC of two variables.

### Highly Discrepant Features Extraction

The selection of appropriate features has a crucial impact on the classification results. However, if too many features are introduced, dimensionality becomes a problem and the classifier loses generalization (43, 44). Therefore, before classification, a small number of features with high discrepancy should be selected carefully. Because MIC and eMIC values do not follow Gaussian distributions, Kendall tau rank correlation coefficient was chosen in this paper as a non-parametric hypothesis test for statistical dependence based on the tau coefficient (45, 46). Let (*x*_1_, *y*_1_), (*x*_2_, *y*_2_), (*x*_3_, *y*_3_), …, (*x_n_, y_n_*) be a set of samples of the joint random variables *X* and *Y*. Each pair of samples (*x*_i_, *y*_i_) and (*x_j_, y_j_*), where *i*≠*j*, are concordant if the ranks for both elements agree, that is, if *x_i_* > *x_j_* and *y_i_* > *y_j_* or if *x_i_* < *x_j_* and *y_i_* < *y_j_*. The pair is discordant if *x_i_* > *x_j_* and *y_i_* < *y_j_* or if *x_i_* < *x_j_* and *y_i_* > *y_j_*. The Kendall tau coefficient (τ) is defined as:

(6) $$\tau =\frac{\left(\text{number of concordant pairs}\right)-\left(\text{number of disconcordant pairs}\right)}{n\left(n-1\right)/2}$$

In this study, The Kendall tau coefficient τ was calculated as:

(7) $$\tau =\frac{n_{\text{c}}-n_{\text{d}}}{n_{\text{t}}}$$

where *n*_c_ represents the number of concordant feature pairs, *n*_d_ represents the number of discordant feature pairs, and *n*_t_ represents the number of all feature pairs. For the feature (*X_ij_, y_j_*), *i* = 1, 2, …, *N, X_ij_* corresponded the *i*th feature of the *j*th subject, and *y_j_* corresponded (CN coded as 1, MCI as 2, dementia as 3). Three groups of participants were defined in this study (CN, MCI, and dementia). Features with maximal discrepancy between groups were identified by pairwise comparison of cognitive level across groups. For example, when comparing subjects from the CN group and the MCI group, the Kendall correlation coefficient for the *i*th feature was redefined as:

(8) $$\tau_{i}=\frac{n_{\text{c}}-n_{\text{d}}}{m\times n}$$

where *m* is the number of subject in the CN group, and *n* is the number of subjects from the MCI group. Because we were not interested in the comparison of subjects within the same group, the number of possible combination was *n_t_* = *m* × *n*. Nine groups of features discrepancy were calculated in this paper, using PCC, MIC, and eMIC methods for FC. Each method was used for comparison between CN and MCI, MCI and dementia, and CN and dementia. The absolute value of τ was used as a measure of difference between features and sorted by value. Finally, feature pairs with the top 1% τ were selected for the classification of cognitive performance.

To investigate relationship of cognitive level and FC with high discriminative power in LA, we related FC based on PCC, MIC, and eMIC to cognition. MMSE test was a behavioral assessment of cognitive impairment; thus, cognition was quantified by MMSE scores. The correlation between FC and MMSE scores was studied using Pearson’s correlation on MATLAB.

In addition, to evaluate the performance of features for classification of cognitive performance, leave-one-out cross-validation was used. The Kendall coefficient was calculated on the training data set. In the network graph of FC pairs showing high discrepancy, ROIs showing FCs with high discrepancy with other brain regions were considered as significant ROIs.

### Classification

Classification was done with SVM using highly discrepant features, i.e., by a supervised machine learning method. The training dataset was used to train the classifier and produce category output. The Matlab LIBSVM v3.17^3^ toolkit designed by Professor Chih-Jen Lin et al. from Taiwan University was used in this study. LIBSVM is a practical and effective package for SVM pattern recognition and regression. C-SVM with a linear kernel function was chosen. The other parameters were set to default values. While monitoring the influence of feature quantity on classification results, the number of features considered for classification was increased according to feature discrepancy values (the classifier was first trained with the first feature (with highest τ), then the classifier was trained with the first two features (with highest and second highest τ) and so on, until all features had been selected). Classification accuracy data was based on test data classified with the same number of features as obtained from the training set. In addition, leave-one-out cross-validation was used. In this procedure, for each number of features selected, *N* classifications were done (where *N* corresponds to the number of subjects). The average over the *N* classification accuracies was similar to the classification accuracy for the corresponding feature number in the training data set.

## Results

In this study, highly discrepant linear and non-linear FC was identified using PCC, MIC, and eMIC from resting-state fMRI data from patients with LA. These data were used to analyze abnormality of non-linear and linear FCs in LA. Based on highly discrepant FC, patients with LA were classified into three groups according to the cognitive levels (CN, MCI, and dementia).

### FC Matrices

Functional connectivity matrixes for each participant were calculated using PCC, MIC, and eMIC, evaluating the statistical level of connection between brain regions. FC matrices of LA were 116 × 116 symmetric dependence matrices, based on 116 brain regions from AAL. Values on the diagonal correspond to the relative values of each brain region. The 6,670 non-redundant FC values in each matrix were considered in the feature selection step.

### Brain Regions Showing Significantly Different Functional Connectivity for Different Cognitive Levels

**Figure 1:**
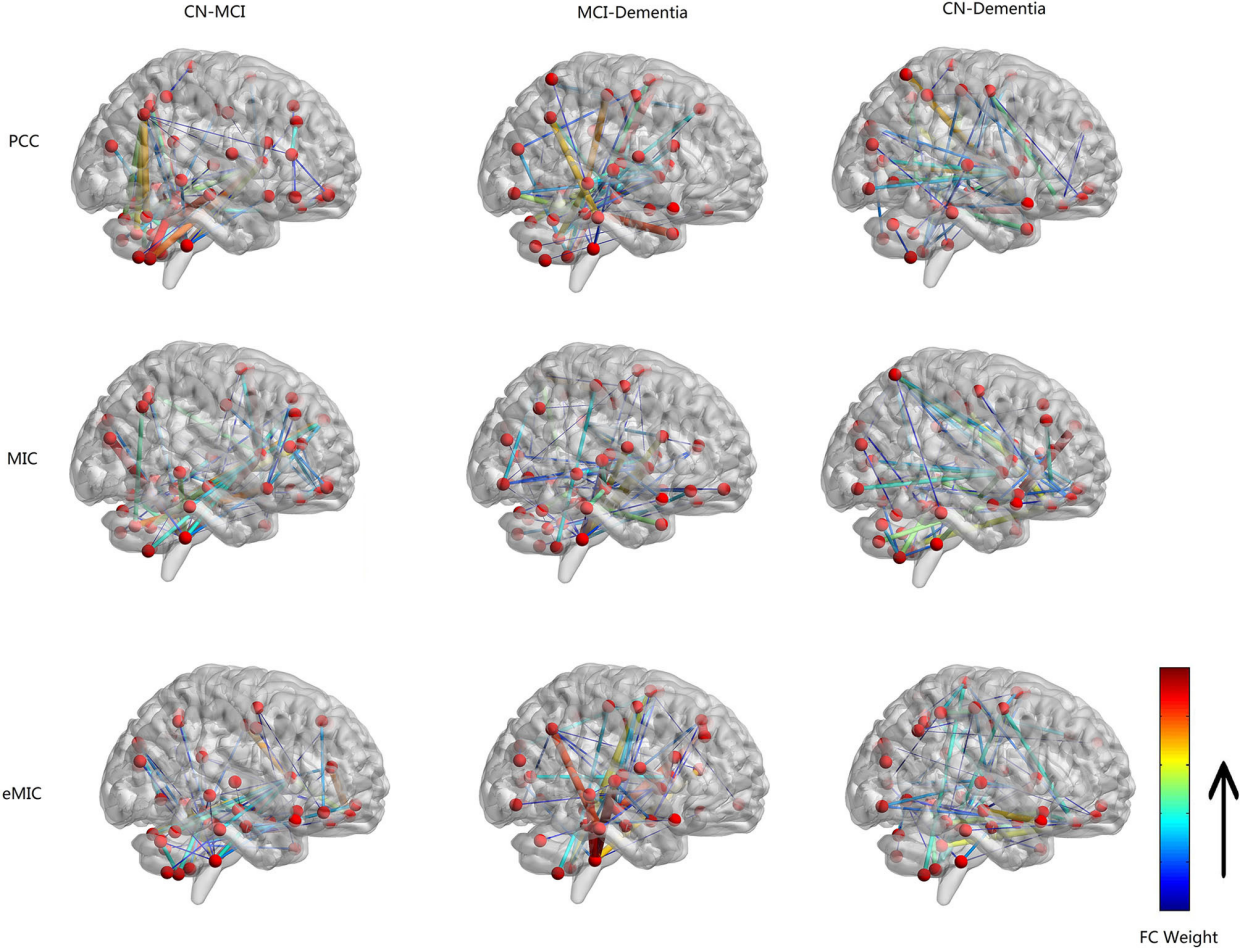
**Network graph of functional connectivity (FC) with high discrepancy based on all subjects**. Nodes correspond to brain regions of automatic anatomical labeling. Edges between different nodes in the graph correspond the FC with high discrepancy. Color and line width of edges represent the weight of difference.

Functional connectivity of LA patients with different cognitive levels (CN vs. MCI, MCI vs. dementia, and CN vs. dementia) was compared using PCC, MIC, and eMIC as measurement methods yielding nine pairwise comparisons. For each group, the FC discrepancy was calculated, and 67 highly discrepant features were extracted (Figure 1), and significant ROIs with between-group connectivity differences were listed (Table 2).

**Table 2:** **Significant ROIs with between-group connectivity differences**.

	CN vs. MCI	MCI vs. dementia	CN vs. dementia
Pearson’s correlation coefficient	Caudate_L	Hippocampus_L	Cerebelum_9_L
	Cerebelum_10_L	Cerebelum_9_L	Insula_L
	Vermis_3	Vermis_3	Insula_R
	Angular_R	Temporal_Mid_R	Temporal_Pole_Sup_L
MIC	Caudate_L	Hippocampus_L	Rectus_R
	Cerebelum_10_L	Cerebelum_10_L	Insula_R
	Vermis_3	Parietal_Sup_L	Parietal_Sup_R
	Frontal_Sup_Orb_L	Putamen_R	Temporal_Pole_Sup_L
eMIC	Caudate_L	Hippocampus_L	Rectus_R
	Cerebelum_10_L	Hippocampus_R	Precentral_L
	Vermis_3	Cerebelum_9_L	Cerebelum_9_L
	Frontal_Sup_Orb_L	Cerebelum_10_R	

The first column of Figure 1 shows the result of the comparison of FC between CN and MCI. Based on highly discrepant FCs calculated by PCC, MIC, and eMIC, significant changes between significant ROIs and other regions were identified. Based on PCC, left caudate nucleus (Caudate_L), left lobule X of cerebellar hemisphere (flocculus; Cerebelum_10_L), lobule III of vermis (Vermis_3), and right angular gyrus (Angular_R) were identified as significant ROIs. Based on MIC, Caudate_L, Cerebelum_10_L, Vermis_3, and left superior frontal gyrus, orbital part (Frontal_Sup_Orb_L) were identified as significant ROIs. Based on eMIC, Caudate_L, Cerebelum_10_L, Vermis_3, and Frontal_Sup_Orb_L were identified as significant ROIs.

The second column of Figure 1 shows the result of the comparison of FC between MCI and dementia. Based on PCC, left hippocampus (Hippocampus_L), left lobule IX of cerebellar hemisphere (Cerebelum_9_L), Vermis_3, and right middle temporal gyrus (Temporal_Mid_R) were identified as significant ROIs. Based on MIC, Hippocampus_L, Cerebelum_10_L, left superior parietal lobule (Parietal_Sup_L), and right putamen (Putamen_R) were identified as significant ROIs. Based on eMIC, Hippocampus_L, right hippocampus (Hippocampus_R), Cerebelum_9_L, and right lobule X of cerebellar hemisphere (flocculus; Cerebelum_10_R) were identified as significant ROIs.

The third column of Figure 1 shows the result of the comparison of FC between CN and dementia. Based on PCC, Cerebelum_9_L, left and right insula (Insula_L and Insula_R), and left superior temporal pole (Temporal_Pole_Sup_L) were identified as significant ROIs. Based on MIC, right gyrus rectus (Rectus_R), Insula_R, right superior parietal lobule (Parietal_Sup_R), and Temporal_Pole_Sup_L were identified as significant ROIs. Based on eMIC, Rectus_R, left precentral gyrus (Precentral_L), and Cerebelum_9_L were identified as significant ROIs.

**Figure 2:**
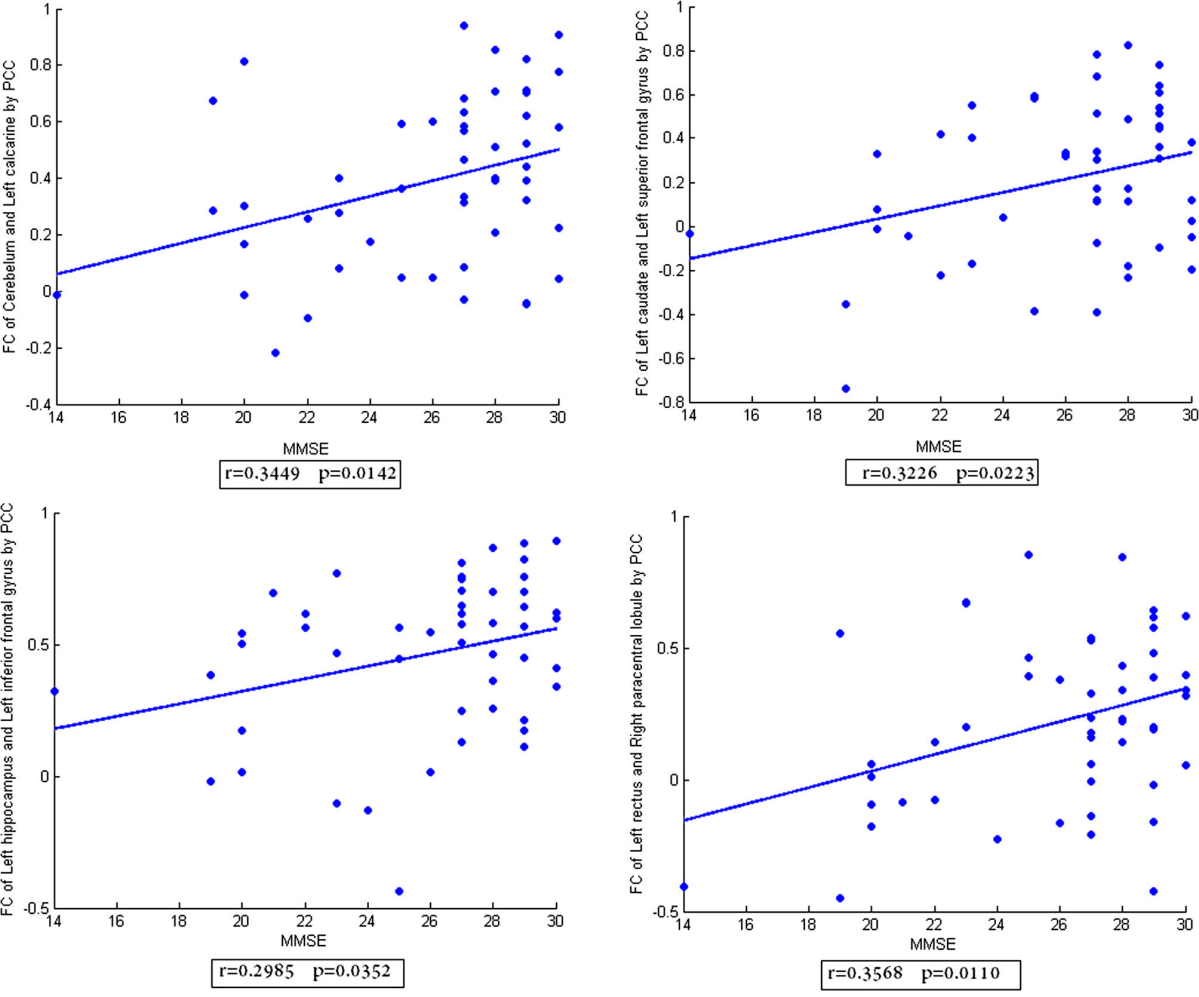
**The scatter plot of functional connectivity (FC) calculated by Pearson’s correlation coefficient vs. the mini-mental state examination scores**. The lines in each subplot show the linear regression. The correlation (*r*) and *p*-value (*p*) for each FC were added to the corresponding subplot.

**Figure 3:**
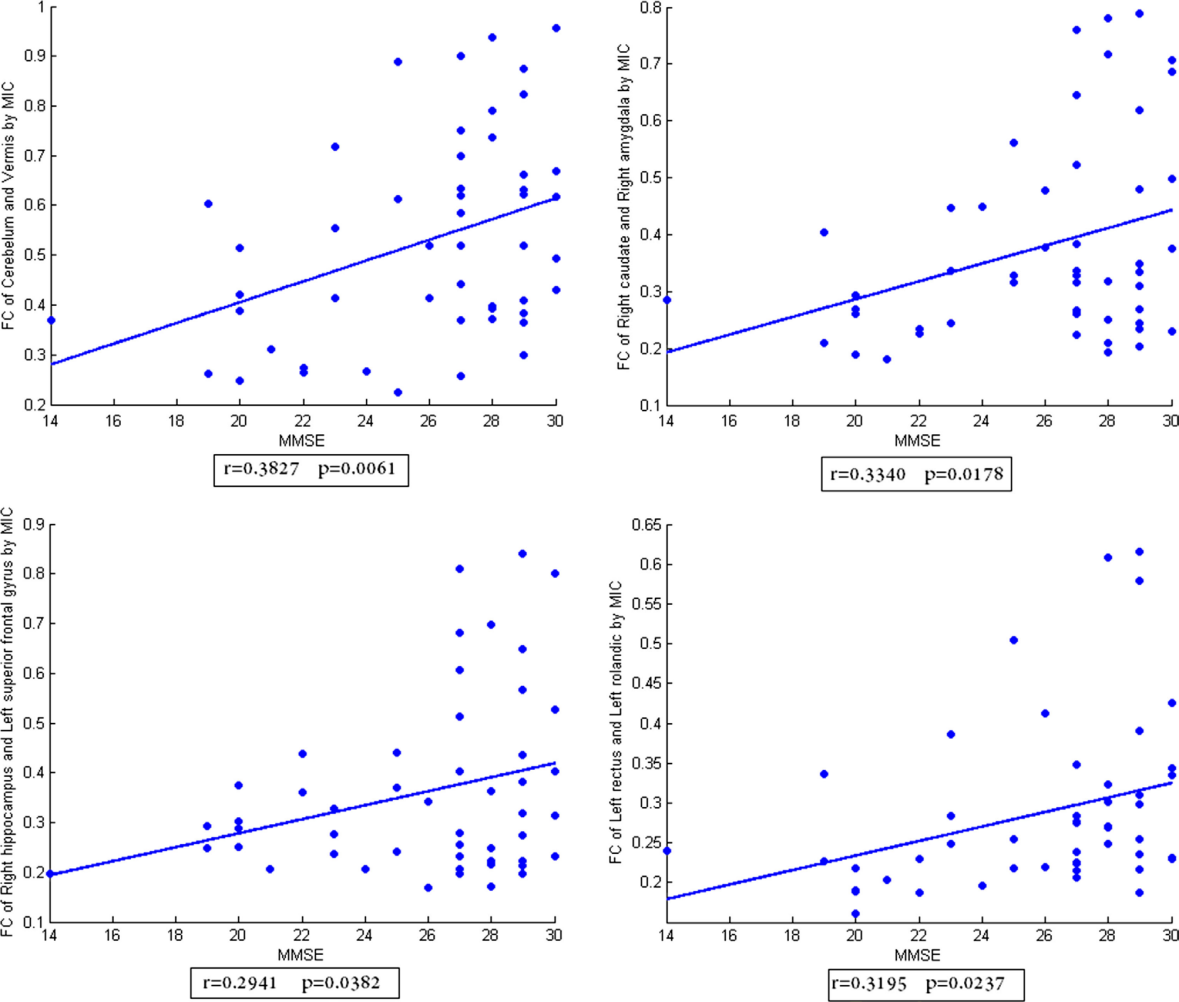
**The scatter plot of functional connectivity (FC) calculated by MIC vs. the mini-mental state examination scores**. The lines in each subplot show the linear regression. The correlation (*r*) and *p*-value (*p*) for each FC were added to the corresponding subplot.

**Figure 4:**
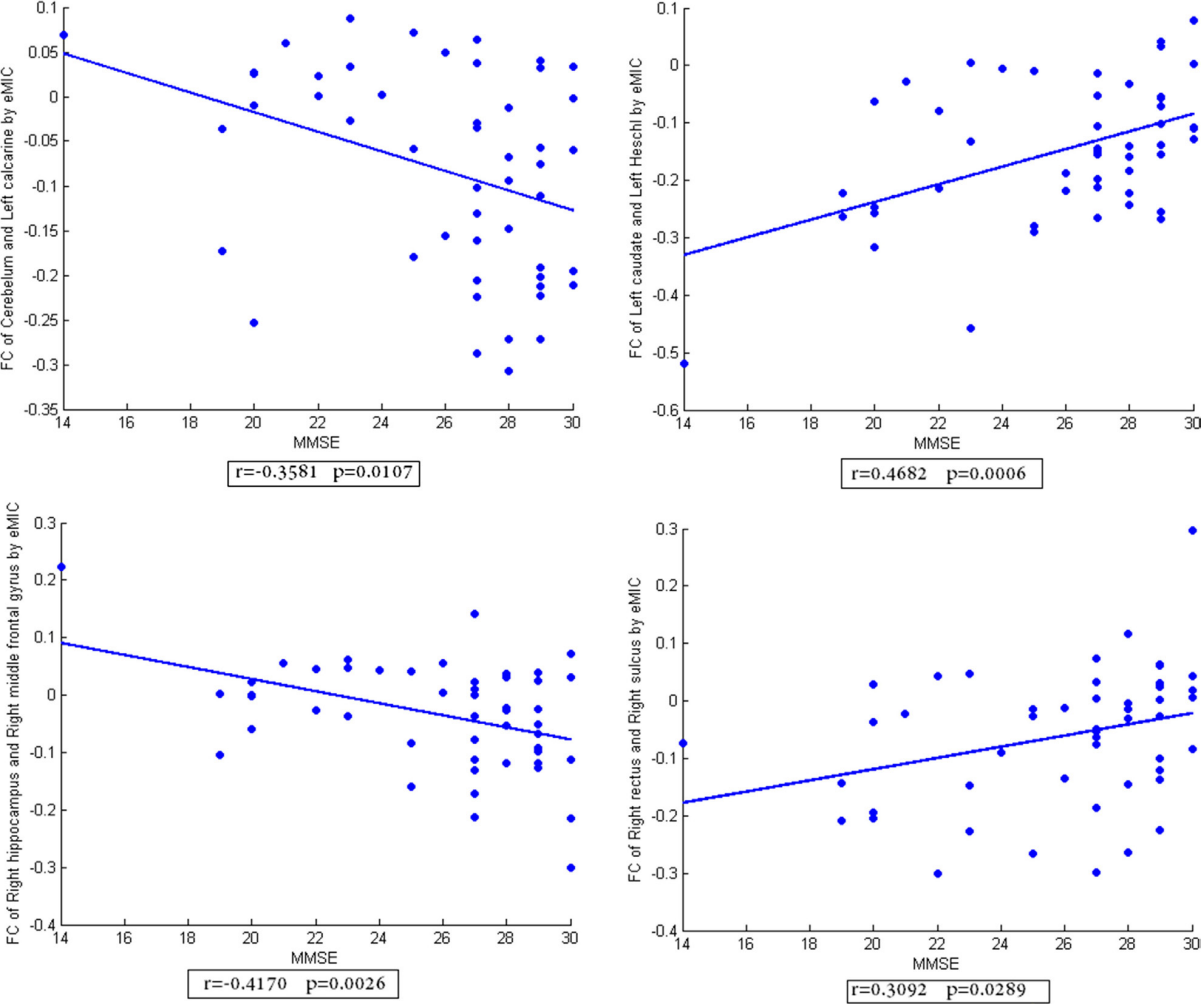
**The scatter plot of functional connectivity (FC) calculated by eMIC vs. the mini-mental state examination scores**. The lines in each subplot show the linear regression. The correlation (*r*) and *p*-value (*p*) for each FC were added to the corresponding subplot.

In addition, the scatter plots of FC vs. MMSE scores were done (Figures 2–4). Here, the main results with significant (*p* < 0.05) positive or negative correlation were list, including the relationship between MMSE- and PCC-based FC of Cerebelum and Left calcarine, Left caudate and Left superior frontal gyrus, Left hippocampus and Left inferior frontal gyrus, Left rectus and Right paracentral lobule (Figure 2), the relationship between MMSE and MIC-based FC of Cerelelum and Vermis, Right caudate and Right amygdala, Right hippocampus and Left superior frontal gyrus, Left rectus and Left rolandic (Figure 3), the relationship between MMSE and eMIC-based FC of Cerelelum and Left calcarine, Left caudate and Left heschl, Right hippocampus and Right middle frontal gyrus, Right rectus and Right sulcus (Figure 4).

### Classification Result

**Figure 5:**
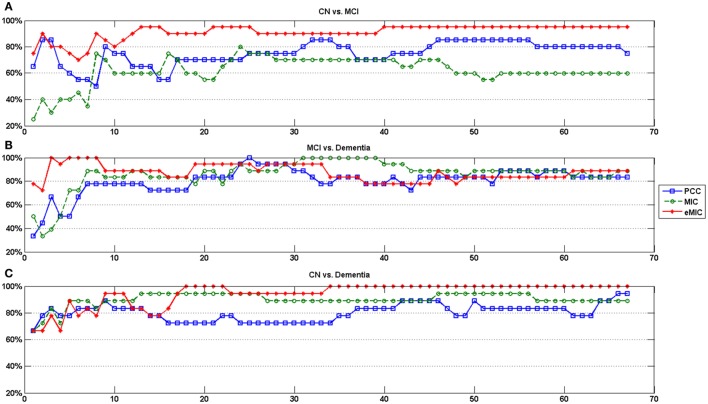
**Classification accuracy of LA cognitive performance with support vector machine**. Horizontal axes show the number of features in the classification. **(A)** The classification accuracy of CN and MCI. **(B)** The classification accuracy of MCI and dementia. **(C)** The classification accuracy of CN and dementia.

**Figure 6:**
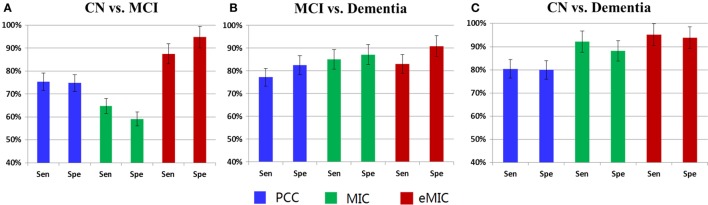
**Sensitivity and specificity of LA cognitive level classification (Sen, sensitivity; Spe, specificity)**. **(A)** The sensitivity and specificity for classification of CN and MCI. **(B)** The sensitivity and specificity for classification of MCI and dementia. **(C)** The sensitivity and specificity for classification of CN and dementia.

We calculated the classification accuracy based on different numbers of features (Figure 5). The average classification accuracy for different numbers of features was used as the classification accuracy for CN vs. MCI, MCI vs. dementia, and CN vs. dementia (Table 3). In order to obtain a more comprehensive evaluation of the classification result, sensitivity and specificity of classification results were calculated (Figure 6).

**Table 3:** **Average classification accuracy of LA cognitive performance with SVM**.

	CN vs. MCI	MCI vs. dementia	CN vs. dementia
Pearson’s correlation coefficient (%)	75.0	80.1	80.1
MIC (%)	61.9	86.2	89.9
eMIC (%)	91.1	87.4	94.4

The first panel of Figure 5 depicts the classification accuracy of CN and MCI based on different numbers of features. Highest classification accuracy was obtained using features calculated by eMIC. In contrast, lowest classification accuracy was obtained using features calculated by MIC. When the feature count was less than 10, classification accuracy was unstable and relatively low. As feature number increased, classification accuracy became more stable. The second panel of Figure 5 depicts the classification accuracy of MCI and dementia based on different numbers of features. Again, classification accuracy was unstable, when the feature count was less than 10. However, classification accuracy was not significant different for features calculated from PCC, MIC, and eMIC. The third panel of Figure 5 depicts the classification accuracy of CN and dementia. Again, classification accuracy was lower when classification was based on less than 10 features. Classification accuracy was higher when based on features from eMIC than from PCC and MIC. Classification accuracy was the lowest when based on features calculated from PCC.

The average classification accuracy was calculated based on data presented in Figure 5. The average classification accuracy obtained from each method was defined as the average of all classification accuracies calculated from different numbers of selected features (Table 3). We found that average classification accuracy for MCI and dementia based on features obtained from eMIC was 91.1%, which was higher than that based on features obtained from MIC (61.9%). Average classification accuracy based on features obtained from PCC was 75.0%. For classification of MCI and dementia, PCC yielded the lowest average classification accuracy (80.1%). Classification based on features obtained from MIC and eMIC yielded similar average classification accuracy of 86.2 and 87.4%, respectively. Average classification accuracy for CN and dementia was 81.0% for PCC, which was lower than that for features from MIC (89.9%) and eMIC (94.4%).

For classification of CN and MCI (first panel of Figure 6), sensitivity and specificity of classification with features obtained from PCC were 75.4 and 74.7%, respectively, for features obtained from MIC were 64.7 and 59.1%, respectively, and for features obtained from eMIC were 87.4 and 94.7%, respectively. Regarding classification of MCI and dementia (second panel of Figure 6), sensitivity and specificity of classification with features obtained from PCC was 77.2 and 82.5%, respectively, for features obtained from MIC were 85.0 and 87.1%, respectively, and from eMIC 83.0 and 90.9%, respectively. For CN and dementia (third panel of Figure 6), sensitivity and specificity for classification with features obtained from PCC were 80.4 and 80.0%, respectively, from MIC 92.1 and 88.0%, respectively, and from eMIC 95.1 and 93.8%, respectively.

## Discussion

In this study, accurate cognitive performance classification of patients with LA was achieved based on linear and non-linear FC. There were two main results in this study. First, we found that FC differed between patients with CN, MCI, and dementia: FC significantly differed in the left caudate nucleus and the left inferior cerebellum for patients with MCI and patients with CN; FC differed in the bilateral hippocampus for patients with dementia and patients with MCI; and FC differed in the right rectus for patients with dementia and patients that were CN. Second, when cognitive level of patients with LA was estimated based on FC, classification based on eMIC was most accurate and stable. Classification accuracy based on eMIC was 91.1% for CN and MCI, 87.4% for MCI and dementia, and 94.4% for CN and dementia.

### Difference of FC in LA

The correlation results between MMSE scores of subjects and FC calculated by PCC, MIC, eMIC show that the FC in specific brain regions had significant (*p* < 0.05) positive or negative correlation with the MMSE scores. The strength of PCC- and MIC-based FC was significant decreased with the decline of cognitive level in LA, which was consistent with the common view (12, 13, 23–26). In contrast, a part of eMIC-based FC demonstrated increased strength with the decline of cognitive level, such as the FC of left caudate and left heschl, right rectus and right sulcus. This shows the compensatory mechanism in human brain, that is, the non-linear FC would strengthen to compensate the breakdown of linear FC.

A similar distribution of highly discrepant FC was obtained for significant ROIs when FC discrepancy was calculated using PCC, MIC, and eMIC. In patients with LA, FC between the cerebellum and other brain regions significantly differed across patients with CN, MCI, and dementia, in particular with left calcarine, vermis, right middle temporal gyrus, and amygdala, middle temporal pole. Several previous studies have shown that lesions of the cerebellum were related to clinically significant disturbances of language, behavior, and cognitive level (47–49). The change in FC of the cerebellum with other brain regions suggests that dysfunctions observed in relation to cerebellar lesions might reflect a dysfunction in communication with other brain regions.

In addition, results of FC distribution with significant differences suggest that in the early period of cognitive impairment, namely CN vs. MCI, FC differed in the caudate nucleus and left inferior cerebellum. However, FC in the bilateral hippocampal region, an important region of dementia, changed significantly. Ryan et al. reported reduced volume of the bilateral caudate nucleus and increased fractional anisotropy of bilateral thalamus and left caudate nucleus at a presymptomatic stage of Alzheimer’s disease (50). The caudate nucleus plays a key role in planning and carrying out complex behavior (51, 52). In contrast, several other studies did not report significant differences between MCI and healthy controls in hippocampal regions. Johnson et al. and Ries et al. found no evidence for activation differences in hippocampal regions between MCI and controls in retrieval tasks (53, 54). In addition, Zhou et al. did not observe decreased FC for MCI when compared with matched controls (55). In conjunction with findings from previous studies, our results may indicate that the level of dysfunction is more critical resulting in impairment of executive abilities, rather than memory disturbances evident from functional changes in the hippocampal regions in early period of cognitive impairment in LA (56).

When MCI was compared with dementia, FC of bilateral hippocampus had highly discrepant FCs. In LA, when patients developed dementia, obvious memory disorders were observed (57). Bilateral hippocampus is the main region processing short-term memory in the brain (58). In addition, previous studies have shown that the degree of memory loss depended on the extent of hippocampal removal (59). Changes in FC of the bilateral hippocampus might aid in explaining the occurrence of memory disorders in dementia.

When CN was compared with dementia, the rectus was highly discrepant FCs compared with other regions. In a study of patients with LA who had urinary incontinence, Kuchel et al. found that the presence of FC in right inferior frontal regions and selected white matter tracts could predict incontinence, incontinence severity, and degree of bother (60). The right rectus forms part of the prefrontal cortex. A previous study of several types of dementia, including frontotemporal dementia, vascular dementia, and AD, showed that it significantly differed from CN (61–63), suggesting that the rectus might be a brain region significantly associated with cognitive level, but not sensitive to this specific type of dementia.

### Classification Result Based on the Linear and Non-Linear FC

Classification of cognitive level-based eMIC (non-linear FC) achieved the highest classification accuracy when compared with results from classification based on PCC and MIC. In addition, eMIC combines the advantages of PCC and MIC, exhibiting the high classification accuracy inherent to MIC while and benefiting from the stability of PCC. Linear FC is often used to study the communication of information between different brain regions. However, non-linear FC has been shown to be more effective to study interactions between brain regions, because the brain is a complex system, and non-linear relationships between the activity of different brain regions is to be expected (34, 64, 65). eMIC, which has been used to evaluate non-linear relationships between two brain regions, captures subtle changes in FC and uses more discriminative information for classification (36). The results of the present study indicate that for the analysis of different levels of cognitive impairment in LA, eMIC-based FC (capturing non-linear dependencies) provides more information and is more effective than other measures.

In addition, the classification of CN and dementia based on PCC, MIC, and eMIC was significantly more successful that classifying CN and MCI, and MCI and dementia. The level of cognitive impairment between CN and dementia is obviously different, and brain changes associated with dementia, such as structure atrophy, reduced functional activation, are very common. Therefore, classification of groups with such obvious differences is easy (66, 67).

## Conclusion

In this study, non-linear and linear FC based on PCC, MIC, and eMIC were used to classify cognitive level of patients with LA. Comparison of classification results showed that features obtained from non-linear FC were superior to features obtained from linear FC when classifying the cognitive level of patients with LA. Moreover, classification results based on non-linear characteristics behaved more balanced than those based on linear characteristics. Therefore, non-linear FC may help to develop more accurate and reasonable clinical classification of cognitive impairment. This article may spur further study of how linear and non-linear information can be used to investigate LA with different cognitive impairment levels. This research enriches neuroimaging data processing and analysis methods and can help provide tools for clinical diagnosis and treatment.

## Author Contributions

XW and RL designed and wrote the article; YZ carried out the experiment and collected the data; RL and YL analyzed the data; LY participated in the discussion and criticized the manuscript.

## Conflict of Interest Statement

The authors declare that the research was conducted in the absence of any commercial or financial relationships that could be construed as a potential conflict of interest.

## Footnotes

^1^http://www.fil.ion.ucl.ac.uk/spm.

^2^http://www.ansir.wfubmc.edu.

^3^http://www.csie.ntu.edu.tw/~cjlin/libsvm/.
